# Human herpesvirus type 6 reactivation after haploidentical hematopoietic cell transplantation with post-transplant cyclophosphamide and antithymocyte globulin: risk factors and clinical impact

**DOI:** 10.46989/001c.92525

**Published:** 2024-02-02

**Authors:** Annalisa Paviglianiti, Tânia Maia, Joël-Meyer Gozlan, Eolia Brissot, Florent Malard, Anne Banet, Zoé Van de Wyngaert, Tounes Ledraa, Ramdane Belhocine, Simona Sestili, Antoine Capes, Nicolas Stocker, Agnès Bonnin, Anne Vekhoff, Ollivier Legrand, Mohamad Mohty, Rémy Duléry

**Affiliations:** 1 Hematology Sorbonne University; 2 Università Campus Bio-Medico; 3 Clinical Hematology Institut Català d’Oncologia; 4 Clinical Hematology and Cellular Therapy, Hôpital Saint-Antoine, Assistance Publique - Hôpitaux de Paris; 5 Clinical Hematology Hospital de São João; 6 Virology Sorbonne University; 7 Virology, Hôpital Saint-Antoine, Assistance Publique - Hôpitaux de Paris; 8 INSERM, UMRs 938 Centre de Recherche Saint-Antoine

**Keywords:** Human herpesvirus type 6, Haploidentical hematopoietic cell transplantation, Antithymocyte globulin, Graft-versus-host disease, Immune reconstitution

## Abstract

Human herpesvirus type 6 (HHV6) reactivation after haploidentical hematopoietic cell transplantation (HCT) with post-transplant cyclophosphamide (PT-Cy) has been scarcely studied, especially when antithymocyte globulin (ATG) is added to the graft-versus-host disease (GvHD) prophylaxis. We conducted a retrospective cohort study in 100 consecutive patients receiving haploidentical HCT with PT-Cy. We systematically monitored HHV6 DNA loads in blood samples on a weekly basis using quantitative PCR until day +100. The 100-day cumulative incidence of HHV6 reactivation was 54%. Clinically significant HHV6 infections were rare (7%), associated with higher HHV6 DNA loads, and had favorable outcomes after antiviral therapy. The main risk factor for HHV6 reactivation was a low absolute lymphocyte count (ALC) < 290/µL on day +30 (68% versus 40%, p = 0.003). Adding ATG to PT-Cy did not increase the incidence of HHV6 reactivation (52% with ATG versus 79% without ATG, p = 0.12). Patients experiencing HHV6 reactivation demonstrated delayed platelet recovery (HR 1.81, 95% CI 1.07-3.05, p = 0.026), higher risk of acute grade II-IV GvHD (39% versus 9%, p < 0.001) but similar overall survival and non-relapse mortality to the other patients. In conclusion, our findings endorse the safety of combining ATG and PT-Cy in terms of the risk of HHV6 reactivation and infection in patients undergoing haploidentical HCT. Patients with a low ALC on day +30 face a higher risk of HHV6 reactivation and may require careful monitoring.

## INTRODUCTION

Human herpesvirus type 6 (HHV6) is a common virus, with a seroprevalence of around 95% in the general population. Two variants of the virus, type A and type B, have been identified, which exhibit distinct biological characteristics and are associated with different diseases. Infection typically occurs in early childhood.^1,2^ While HHV6 primarily infects CD4+ T-cells, it can also establish a persistent infection in various other cell types, including mononuclear cells, salivary glands, brain tissue, lymph nodes, and endothelial cells. In rare cases, the virus is also capable of chromosomal integration, which is a unique mechanism of viral latency, a phenomenon observed in approximately 1% of the population.^3^ HHV6, particularly variant B, can reactivate and cause opportunistic diseases in patients under immunosuppressive treatment.^4^ In the context of allogeneic hematopoietic cell transplantation (HCT), HHV6 reactivation arises in 47% to 72% of patients, posing a significant risk of morbidity and mortality.^5–9^ In particular, HHV6 stands as the foremost cause for viral encephalitis in this population.^10,11^

Previous studies have evaluated risk factors and subsequent effects of HHV6 reactivation in HCT recipients, especially in umbilical cord blood transplant (UCBT), where HHV6 reactivation can reach an incidence of 80% to 97%, due to the naive status of infused T-cells and delayed hematopoietic and immune reconstitution.^12,13^ However, data on HHV6 reactivation after haploidentical (HAPLO) HCT are limited, especially in the case of high-dose post-transplant cyclophosphamide (PT-Cy) administration.^14–18^ Furthermore, most studies have involved small series and did not include rabbit antithymocyte globulin (ATG) as part of the graft-versus-host disease (GvHD) prophylaxis. Indeed, combining PT-Cy with ATG has recently emerged as a promising strategy to further reduce the risk of GvHD in HAPLO HCT with a peripheral blood stem cell (PBSC) graft.^19–23^ Retrospective studies suggest that this combination may result in a lower incidence of chronic GvHD without increasing the risks of relapse, when compared to PT-Cy without ATG. However, there are concerns that adding ATG might increase the risk of viral reactivations, especially cytomegalovirus (CMV), or lead to an increase in Epstein-Barr virus (EBV) viral loads.^24^ To date, no study has specifically assessed the impact of adding ATG to PT-Cy on HHV6 reactivation. Therefore, we aimed to evaluate the cumulative incidence and the risk factors of HHV6 reactivation, as well as its impact on clinical outcomes in a HAPLO setting when PT-Cy is combined with low-dose ATG.

## MATERIALS AND METHODS

### Study Population and Transplantation Modalities

This is a retrospective study evaluating all consecutive adult patients who underwent HAPLO HCT with PT-Cy at Saint-Antoine Hospital in Paris, France, between January 2013 and December 2017. HHV6 monitoring was conducted systematically during this period at our center. All patients who proceeded to transplantation provided written informed consent for the use of their data in clinical research, in accordance with the modified guidelines of the Declaration of Helsinki and the local institutional review board.

### Conditioning regimens, GvHD prophylaxis, and transplantation modalities

The majority of patients received a conditioning regimen including thiotepa, as previously published.^25,26^ A total busulfan dose of 9.6 mg/kg was considered as myeloablative conditioning (MAC). Sequential conditioning consisted of a short course of intensive chemotherapy followed by a reduced intensity conditioning (RIC) regimen. Other conditioning regimens were considered as RIC. Stem cell sources included PBSC or bone marrow (BM).

GvHD prophylaxis consisted of cyclosporine A, mycophenolate mofetil (MMF), and PT-Cy for all patients. Cyclosporine A was initiated at a dose of 3 mg/kg/day and gradually tapered, starting from days +60 to +90 (depending on disease risk and GvHD history), while MMF was given at a dose of 30 mg/kg/day until day +35. Patients could receive rabbit ATG (Thymoglobulin, Sanofi, Paris, France) at a dose of 2.5 mg/kg/day on day -2 (in MAC or RIC) or on days -3 and -2 (mostly in sequential conditioning). High-dose PT-Cy was administered at a dose of 50 mg/kg/day on days +3 and +5 in PBSC recipients. BM recipients were scheduled to receive a single PT-Cy dose of 50 mg/kg on day +3.

Infection prophylaxis consisted of amoxicillin, trimethoprim-sulfamethoxazole (or atovaquone), and valacyclovir (or acyclovir). Patients were systematically monitored for CMV and aspergillosis twice a week, and for HHV6, EBV, BK virus, and *Toxoplasma gondii* once a week until at least day +100 , as previously reported.^17,27,28^

### Detection of HHV6

Blood samples from patients were collected and subjected to HHV6 real-time polymerase chain reaction (PCR) weekly during the first 100 days after HAPLO HCT. After day +100, the frequency of blood collections depended on the patient´s clinical status, biological signs, and previous HHV6 PCR results. In the event of HHV6 reactivation, HHV6 DNA loads were monitored until they became lower than 500 international units (IU) per mL, as recommended.^29^ HHV6 PCR was systematically performed on gut biopsies in case of colitis and/or suspicion of GvHD. Using the automatized Qiagen (Les Ulis, France) platform, a commercially available real-time PCR kit (Altona Real-Star HHV6 PCR Kit, Jouay-lès-Tours, France) was used to simultaneously detect, discriminate, and quantify the 2 HHV6 subtypes (A and B). The test uses two standard curves generated with four quantitation standards containing 500,000, 50,000, 5,000, and 500 IU of viral DNA per reaction, respectively. DNA was extracted automatically from 200 μL of EDTA-treated whole blood on the Qiasymphony SP automat, using the Qiasymphony DSP DNA Mini Kit (Qiagen). Results are expressed as HHV6 IU/mL of whole blood, with a sensitivity threshold of 50 IU/mL and a quantitation threshold of 500 IU/mL. CMV DNA quantitation was systematically performed in parallel, using the Artus CMV QS-RGQ kit (Qiagen), on the same real-time PCR platform.

### Definitions and Statistical Analysis

HHV6 reactivation was defined as the detection of at least 500 IU/mL HHV6 DNA in peripheral blood in two consecutive samples. According to the European Conference on Infections in Leukaemia (ECIL) guidelines,^29^ a clinically significant HHV6 infection was defined as the presence of HHV6-related end-organ disease (such as encephalitis, pneumonitis, hepatitis, and myelosuppression/allograft failure) or colitis (when biopsy-proven detection of HHV6 DNA was present), in cases where no other causes could explain the symptoms. CMV reactivation was defined as the detection of at least 1,000 IU/mL CMV DNA on peripheral blood in two consecutive samples, which systematically led to preemptive antiviral therapy. Neutrophil engraftment was defined as an absolute neutrophil count > 0.5 x 10^9^/L for three consecutive days (the first of which was considered the engraftment day) and platelet engraftment as a platelet count > 50 x 10^9^/L for seven consecutive days (the first of which was considered engraftment day).^30^ Disease type and disease status at the time of transplantation were classified according to disease risk index (DRI).^31^ Overall survival (OS) was defined as time from HCT until death from any cause, disease free survival (DFS) as the probability of being alive and free of disease at any point in time, relapse incidence (RI) as the time from HCT to relapse of the underlying disease, and non-relapse mortality (NRM) as the time from HCT to death without relapse. GvHD was diagnosed and graded according to standard criteria.^32–34^

Patients’ characteristics were compared using the Mann-Whitney test for continuous variables, and the chi-squared or Fisher’s exact test for categorical variables. Absolute lymphocyte counts (ALC) were compared at days +30, + 100, and +180 after HAPLO HCT according to HHV6 reactivation using the Wilcoxon-Mann-Whitney test. Cumulative incidence was used to estimate the occurrence rates of various endpoints, including HHV6 and CMV reactivations, neutrophil and platelet engraftment, GvHD, relapse, and NRM, to accommodate competing risks. The competing events considered were relapse and death for GvHD, relapse for NRM, and death for infectious complications. Probabilities of OS and DFS were calculated using the Kaplan–Meier method. Univariate analyses were performed using Gray’s test for cumulative incidence functions and the log-rank test to compare survival. Variables with a p value < 0.10 were introduced into a multivariate model. Multivariate analyses were performed using Cox’s proportional-hazards regression model for DFS and OS, and Fine and Gray’s proportional-hazards regression model for GvHD, NRM, and RI. On multivariate analysis, the impact of HHV6 reactivation was studied as a time-dependent variable. P values were two-sided, and differences were considered to be significant when the p value was < 0.05. The confidence interval (CI) was set at 95%. Analyses were performed with SPSS 20 (IBM SPSS Statistics for Windows, Version 20.0. IBM Corp, Armonk, NY) and R 3.5.0 (R Foundation for Statistical Computing, Vienna, Austria) software packages.

## RESULTS

### Patient Characteristics and HHV6 reactivation

**Figure 1.**
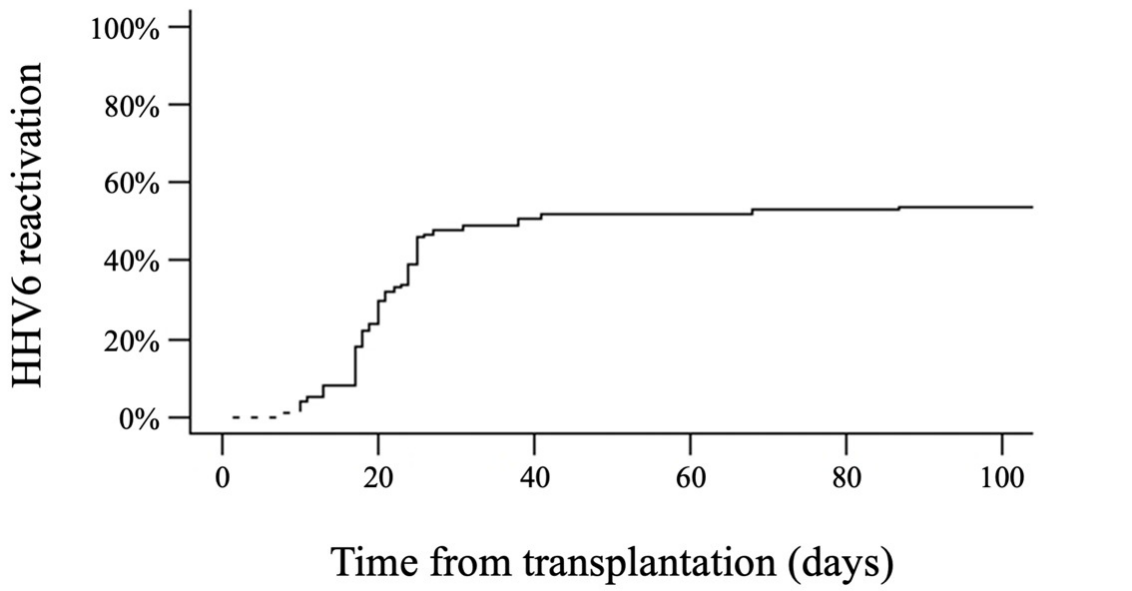
Cumulative incidence of HHV6 reactivation after haploidentical hematopoietic cell transplantation with post-transplant cyclophosphamide (at day +30: 48%, at day +100: 54%).

Patient and disease characteristics are summarized in ***Table 1***. Overall, 1,679 HHV6 PCR tests were performed on blood samples, with a median of 13 HHV6 tests per patient (range, 2-72). HHV6 reactivation, primarily variant B, occurred in 56% of the patients, with a median onset time of 20 days (range, 8-122) after HAPLO HCT (***Figure 1***). The cumulative incidence of HHV6 reactivation at days +30, +60, +100, and +180 after HCT was 48%, 52%, 54%, and 56%, respectively. The median highest HHV6 DNA load was 6,797 IU/mL (range, 524-1,658,173). Among the group of patients with positive HHV6 PCR results, the majority (49 out of 56, 88%) showed no evidence of HHV6-related disease, although mild symptoms such as fever or rash could occur. Seven patients were considered to have clinically significant HHV6 infection, presenting with HHV6-related end-organ disease, including colitis (n=3), encephalitis (n=2), hepatitis (n=1), and myelosuppression with secondary graft failure (n=1). One of the patients with encephalitis also had a concurrent pneumonitis. In cases of clinically significant HHV6 infection, the median highest HHV6 DNA load was 468,447 IU/mL (range, 10,400-1,658,173) compared to 6,135 IU/mL (range, 524-505,945) IU/mL in patients with no signs of infection (p < 0.001). We did not observe any cases of chromosomal integration of HHV6.

**Table 1.** Patient and transplant characteristics

**Characteristics**	**HHV6 reactivation** n (%)	**No HHV6 reactivation** n (%)	**p-⁠value**
**Patient age** (years)			0.22
≤ 51	31 (55)	19 (43)	
> 51	25 (45)	25 (57)	
**Gender**			0.11
Male	38 (68)	23 (52)	
Female	18 (32)	21 (48)	
**Disease**			0.38
Acute myeloid leukemia	29 (52)	21 (48)	
Acute lymphoblastic leukemia	6 (11)	7 (16)	
MDS/MPN	10 (18)	6 (14)	
Lymphoma	8 (14)	10 (23)	
Multiple myeloma	3 (5)	0 (0)	
**Disease status**			0.76
Complete remission	25 (45)	21 (48)	
No complete remission	31 (55)	23 (52)	
**Disease risk index**			0.05
Low - Intermediate	42 (75)	25 (57)	
High - Very High	14 (25)	19 (43)	
**Number of previous treatment lines**			0.72
0 - 1	26 (46)	22 (50)	
≥ 2	30 (54)	22 (50)	
**Previous transplantation**			0.31
No	37 (66)	35 (80)	
Autologous	10 (18)	4 (9)	
Allogeneic	9 (16)	5 (11)	
**Conditioning regimen**			0.7
Myeloablative	17 (30)	10 (23)	
Reduced intensity	16 (29)	14 (32)	
Sequential	23 (41)	20 (45)	
**Stem cell source**			0.23
Bone marrow	16 (29)	8 (18)	
Peripheral blood	40 (71)	36 (82)	
**Patient CMV serostatus**			0.39
Negative	17 (30)	17 (39)	
Positive	39 (70)	27 (61)	
**PT-Cy total dose**			0.92
50 mg/kg	16 (29)	13 (30)	
100 mg/kg	40 (71)	31 (70)	
**Antithymocyte globulin**			0.12
Yes	45 (80)	41 (93)	
No	11 (20)	3 (7)	

*Abbreviations:* HHV6: human herpesvirus type 6, MDS: myelodysplastic syndrome, MPN: myeloproliferative neoplasm, CMV: cytomegalovirus, PT-Cy: post-transplant cyclophosphamide.

### Risk Factors for HHV6 Reactivation

**Figure 2.**
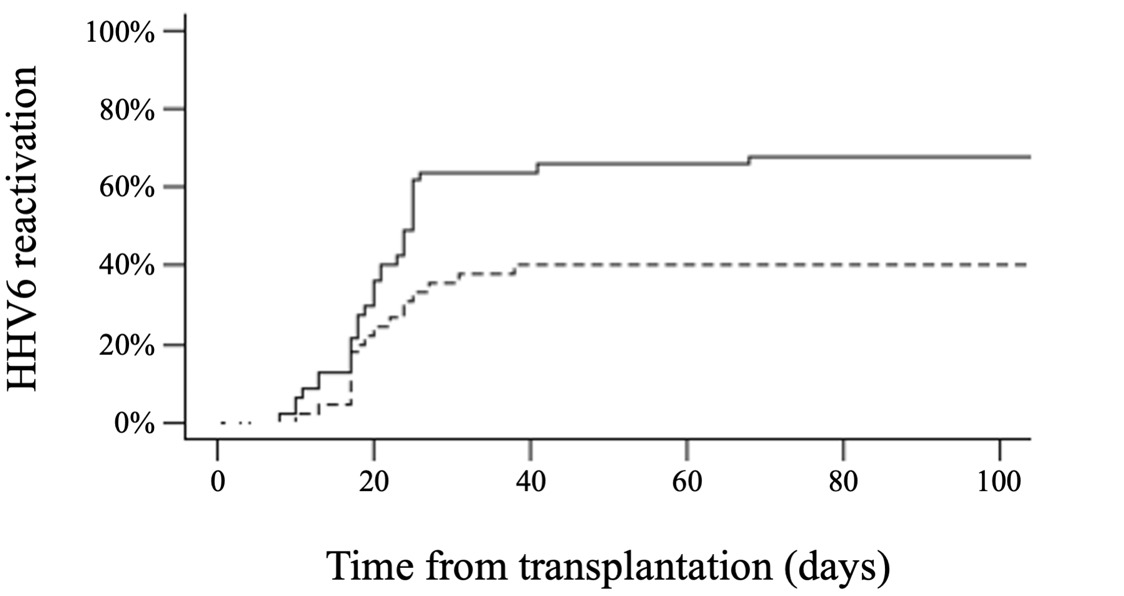
Cumulative incidence of HHV6 reactivation according to the median absolute lymphocyte count (ALC) at day +30 after haploidentical hematopoietic cell transplantation with post-transplant cyclophosphamide (black continuous line: ALC < 290/µL, dashed line ALC ≥ 290/µL). The cumulative incidence of HHV6 reactivation at day +30 was 63% versus 35% (p = 0.003) and, at day + 100, 68% versus 40% (p = 0.003).

At day +30, the median ALC was 290/µL (0-670). Patients with an ALC < 290/µL at day +30 had a higher cumulative incidence of HHV6 reactivation than those with an ALC ≥ 290/µL (63% versus 35%, respectively, p = 0.003) (***Figure 2***). The use of ATG had no significant impact on HHV6 reactivation (52% with ATG versus 79% without ATG, p = 0.12). No other factors such as age, gender, underlying disease, CMV serological status, number of previous treatments, conditioning regimen, or stem cell source were associated with the incidence of HHV6 reactivation.

### Impact on Hematological Engraftment and T-cell Reconstitution

**Figure 3.**
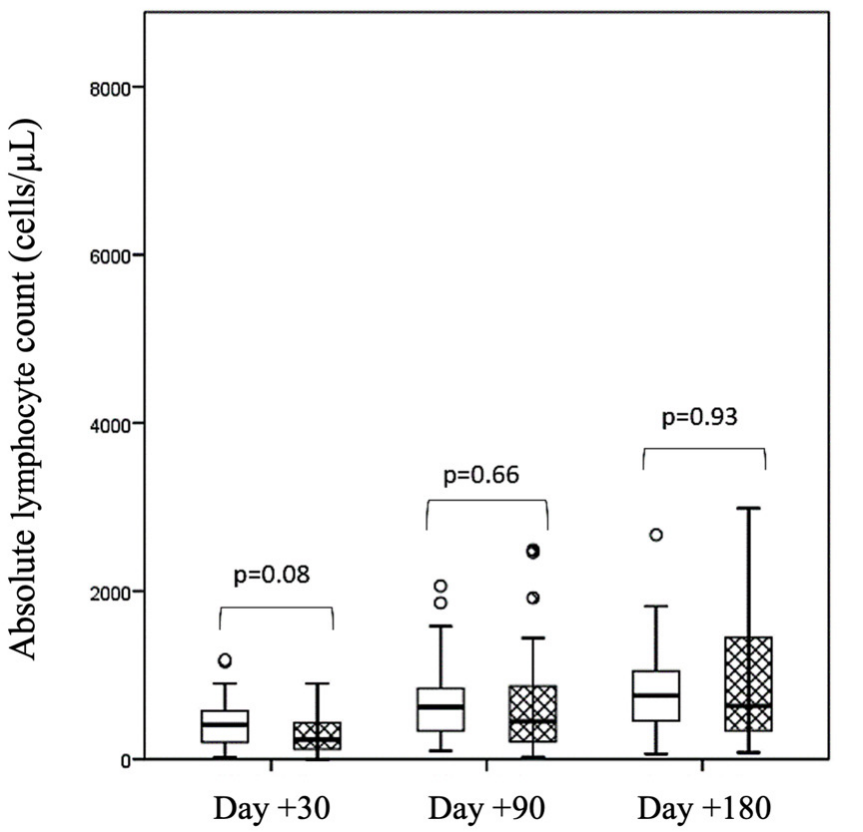
Lymphocyte reconstitution according to HHV6 reactivation at days +30, +90, and +180 after haploidentical hematopoietic cell transplantation with post-transplant cyclophosphamide (grid pattern boxes: HHV6 reactivation, white boxes: no HHV6 reactivation).

At day +30, the cumulative incidence of neutrophil engraftment was 94% (95% CI 88-99). Three patients failed to achieve it. The median time to neutrophil engraftment was 17 days (range, 5-88), and there was no difference according to HHV6 reactivation. Multivariate analysis showed no association between HHV6 reactivation and the cumulative incidence of neutrophil engraftment at day +30 (***Table 2***). At day +180, the cumulative incidence of platelet engraftment > 50 x 10^9^/L was 77% (95% CI 92-100). The median time to platelet engraftment was 33 days (range, 11-373) in cases of HHV6 reactivation and 27 days (range, 13-167) in the absence of HHV6 reactivation. Patients who experienced HHV6 reactivation had a poorer platelet recovery (hazard ratio [HR] 1.81, 95% CI 1.07-3.05, p=0.026) (***Table 2***). Finally, HHV6 reactivation was not significantly associated with the kinetics of lymphocyte reconstitution (***Figure 3***).

**Table 2.** Multivariate analysis of risk factors for HHV6 reactivation

**Cumulative incidence of neutrophil engraftment**	**HR**	**95% CI**	**p value**
HHV6 reactivation* (yes versus no)	1.08	0.60-1.94	0.805
DRI (very high-high versus intermediate-low)	1	0.60-1.56	0.997
Number of previous treatment lines (≥ 2 versus 0-1)	1.11	0.76-1.68	0.631
Patient CMV serostatus (positive versus negative)	0.78	0.51-1.20	0.262
**Cumulative incidence of platelet engraftment**			
HHV6 reactivation* (yes versus no)	1.81	1.07-3.05	0.026
DRI (very high-high versus intermediate-low)	1.68	1.02-2.78	0.043
Number of previous treatment lines (≥ 2 versus 0-1)	0.93	0.59-1.44	0.737
Patient CMV serostatus (positive versus negative)	0.88	0.56-1.44	0.600
**Acute grade II-IV GvHD**			
HHV6 reactivation* (yes versus no)	2.29	0.99-5.27	0.052
DRI (very high-high versus intermediate-low)	1.56	0.69-3.51	0.286
Number of previous treatment lines (≥ 2 versus 0-1)	1.04	0.49-2.22	0.922
Patient CMV serostatus (positive versus negative)	0.95	0.42-2.13	0.891
**Relapse incidence**			
HHV6 reactivation* (yes versus no)	1.44	0.14-14-5	0.759
DRI (very high-high versus intermediate-low)	3.23	1.53-6.79	0.002
Number of previous treatment lines (≥ 2 versus 0-1)	1.66	0.77-3.57	0.195
Patient CMV serostatus (positive versus negative)	1.44	0.67-3.13	0.354
**Non-relapse mortality**			
HHV6 reactivation* (yes versus no)	1.45	0.09-22.5	0.792
DRI (very high-high versus intermediate-low)	1.72	0.77-3.86	0.188
Number of previous treatment lines (≥ 2 versus 0-1)	1.65	0.75-3.63	0.217
Patient CMV serostatus (positive versus negative)	3.13	1.18-8.32	0.022
**Disease-free survival**			
HHV6 reactivation* (yes versus no)	1.52	0.26-8.69	0.641
DRI (very high-high versus intermediate-low)	2.41	1.40-4.15	0.001
Number of previous treatment lines (≥ 2 versus 0-1)	1.64	0.95-2.84	0.079
Patient CMV serostatus (positive versus negative)	2	1.10-3.63	0.02
**Overall survival**			
HHV6 PCR (positive versus negative)	0.69	0.13-3.66	0.665
DRI (very high-high versus intermediate-low)	2.09	1.16-3.76	0.014
Number of previous treatment lines (≥ 2 versus 0-1)	1.81	0.99-3.30	0.054
Patient CMV serostatus (positive versus negative)	2.03	1.05-3.93	0.035

*Abbreviations:* HHV6: human herpesvirus type 6, DRI: disease risk index, CMV: cytomegalovirus, GvHD: graft-versus-host disease, PCR: polymerase chain reaction, HR: hazard ratio.

*HHV6 reactivation was included as a time-dependent risk factor.

### CMV Reactivation

CMV reactivation was observed in 53% of the patients, with a median time of 28 days (range, 3-168) after HCT. The cumulative incidence of CMV reactivation at days +30, +60, +100, and +180 was 29%, 51%, 52%, and 53%, respectively. Co-occurrence of CMV and HHV6 reactivations was observed in 32% of patients (57% of patients with HHV6 reactivation). In only five patients, CMV reactivation preceded HHV6 reactivation. However, a history of HHV6 reactivation was not correlated with a higher incidence of CMV reactivation (p = 0.59). The median time of CMV reactivation occurrence did not significantly differ between patients who experienced HHV6 reactivation and those who did not.

### Impact of Antiviral Treatment on HHV6 Reactivation

Antiviral therapies, including foscarnet, ganciclovir, or cidofovir, were administered to a total of 59 patients, with 37 out of 56 (66%) having HHV6 reactivation. It is important to note that in most patients (31 out of 37), the initiation of antiviral therapy was prompted by concomitant CMV reactivation. Additionally, six patients received antiviral therapy specifically for clinically significant HHV6 infection, such as colitis (n=3), encephalitis (n=2), and hepatitis (n=1). All of these patients had a favorable outcome after undergoing antiviral therapy.

### Graft-versus-Host Disease

**Figure 4.**
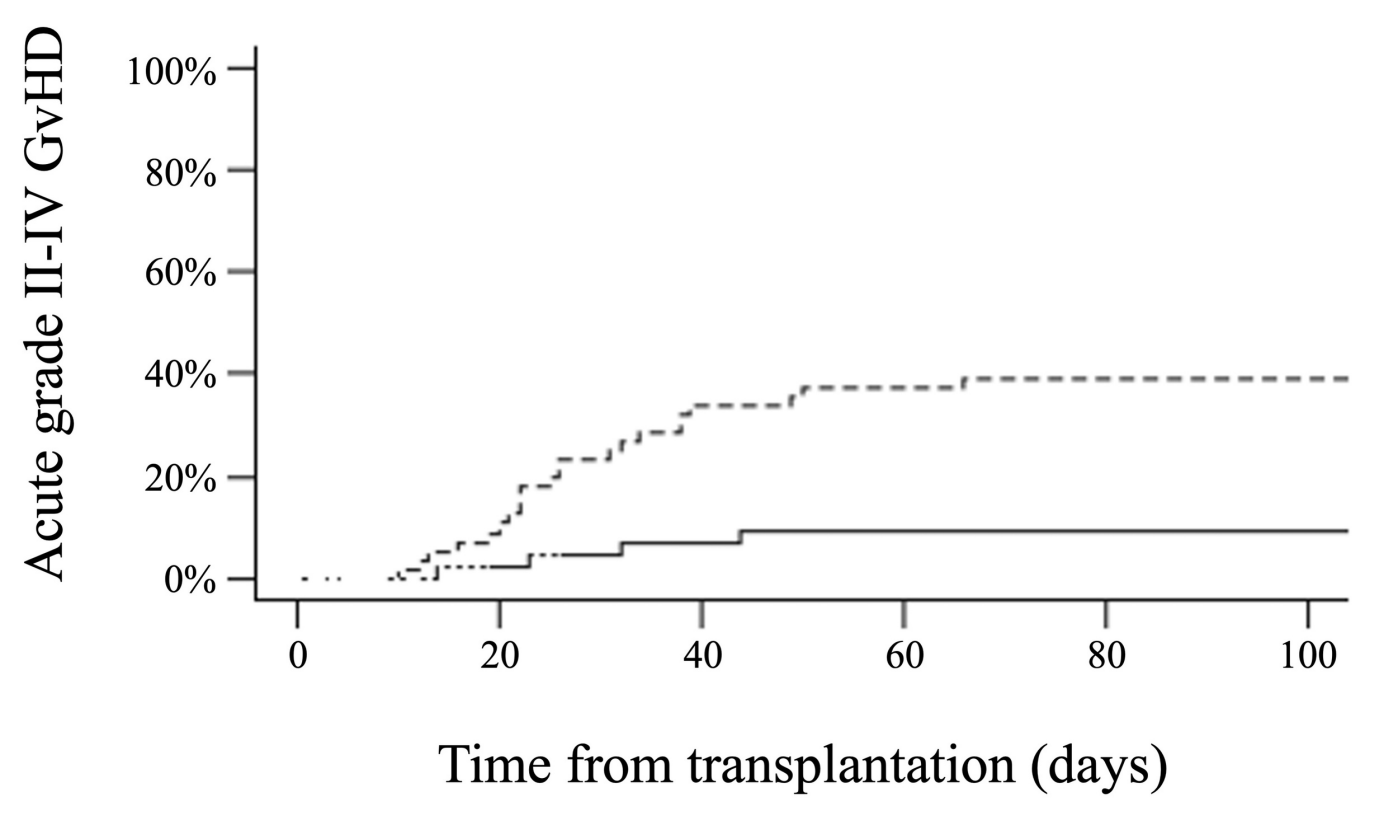
Cumulative incidence of acute grade II-IV GvHD according to HHV6 reactivation within 100 days after haploidentical hematopoietic cell transplantation with post-transplant cyclophosphamide (dashed line: HHV6 reactivation, continuous line: no HHV6 reactivation). The cumulative incidence of acute grade II-IV GvHD at day +100 was 39% versus 9% (p < 0.001).

The cumulative incidence of acute grade II-IV GvHD at day +100 was 26% (95% CI 19-37). In univariate analysis, patients who experienced HHV6 reactivation had a higher cumulative incidence of grade II-IV acute GvHD compared to those who did not (39% versus 9%, p < 0.001) (***Figure 4**, **Supplementary Table S1***). HHV6 reactivation was more frequently observed in patients who experienced acute grade III-IV GvHD, with 12 out of 13 having HHV6 reactivation (p = 0.005). There was an association between a higher HHV6 DNA load and the development of acute grade II-IV GvHD (p = 0.02). However, when HHV6 was analyzed as a time-dependent covariate on multivariate analysis, the association did not reach statistical significance (HR 2.29, 95% CI 0.99-5.27; p=0.05). No association was observed between HHV6 reactivation and the cumulative incidence of chronic GvHD at 1 year (36% in cases of HHV6 reactivation versus 27%, p= 0.36).

### OS, DFS, Relapse, and NRM

After a median follow-up of 36 months (range, 11-76), 48 patients had died. The causes of death included relapse of the underlying hematologic disease (48%), infection (31%), GvHD (10%), hepatic veno-occlusive disease (7%), and miscellaneous (4%). The cumulative incidence of NRM was 12% (95% CI 7-21) at day + 100 and 27% (95% CI 20-38) at 3 years. RI was 29% (95% CI 21-40), OS 51% (95% CI 41-61%), and DFS 44% (95% CI 34-54) at 3 years. On multivariate analysis (***Table 2***), a high or very-high DRI was associated with a higher risk of relapse (HR 3.23, 95% CI 1.53-6.79, p = 0.002), lower OS (HR 2.09, 95% CI 1.16-3.76, p = 0.014), and lower DFS (HR 2.41, 95% CI 1.40-4.15, p = 0.001). A positive CMV serostatus was associated with an increased NRM (HR 3.13, 95% CI 1.18-8.32, p = 0.02), lower OS (HR 2.03, 95% CI 1.05-3.92, p = 0.035), and lower DFS (HR 2.00, 95% CI 1.10-3.63, p=0.02). HHV6 reactivation was not significantly associated with RI, NRM, OS, and DFS (***Table2*** and ***Supplementary Table S1***).

## DISCUSSION

This is one of the largest studies reporting HHV6 reactivation in adult patients undergoing unmanipulated T-cell replete HAPLO HCT with PT-Cy. It is also the first study to assess the incidence, risk factors, and consequences of HHV6 reactivation when pre-transplant low-dose rabbit ATG is added to PT-Cy. Our findings confirm that HHV6 reactivation (mainly variant B) is common in a HAPLO setting with PT-Cy, with a cumulative incidence of 54%. Notably, this incidence is similar to what has been reported with matched related or unrelated donors, and lower than the incidence with UCBT or *ex vivo* T-cell depleted HAPLO HCT. Importantly, the addition of ATG to PT-Cy did not increase the risk of HHV6 reactivation. A key strength of our study is the systematic weekly monitory of HHV6 DNA in blood, a practice which was only implemented in one other study involving HAPLO HCT with PT-Cy.^18^ In the latter, HHV6 reactivation occurred in 112 out of 145 (77%) patients undergoing HAPLO HCT with PT-Cy but without ATG.^18^ In contrast to our study, other reports evaluating HHV6 in this setting have primarily focused on symptomatic reactivations.^14–16^ The lack of systematic monitoring in those studies may underestimate the true incidence of HHV6 reactivation, especially in cases where symptoms were either absent or mild. Regarding clinically significant HHV6 infections, our study demonstrates a low incidence (7%), whereas one study on HAPLO HCT with PT-Cy reported a rate of 16%,^18^ and another analysis of PT-Cy with non-HAPLO donors reported a rate of 24%.^35^ Taken together, our findings suggest the combination of ATG and PT-Cy does not present a higher risk of HHV6 reactivation or clinically significant HHV6 infections compared to PT-Cy without ATG in the HAPLO HCT setting.

Lymphocyte reconstitution is known to be delayed following PT-Cy in HAPLO HCT, and a delay in lymphocyte reconstitution has been associated with a higher incidence of viral reactivations and related diseases, particularly CMV.^36,37^ Combining ATG with PT-Cy can induce a significant reduction in early gamma-delta (γδ) T-cell proliferation, and is associated with a higher risk of increase in EBV viral loads.^38^ However, the impact of such a delayed lymphocyte reconstitution on HHV6 reactivation has scarcely been studied. Two previous studies have reported a protective effect of higher lymphocyte counts (CD3+ lymphocytes ≥ 200/µL) against HHV6 reactivation.^35,39^ Consistent with these results, our analysis revealed that patients with a low ALC on day +30 (< 290/µL) had a higher incidence of HHV6 reactivation. This finding may serve as a valuable tool to help clinicians identify patients at higher risk of HHV6 reactivation, for whom a closer monitoring of HHV6 by PCR might be considered. The low incidence of clinically significant HHV6 infection, the favorable outcome of these infections, and the absence of significant impact of HHV6 reactivation on survival outcomes in our study do not warrant the routine weekly monitoring of HHV6 in all patients undergoing HAPLO HCT, even when ATG is added to the GvHD prophylaxis. In accordance with the ECIL guidelines,^29^ HHV6 PCR is recommended for the diagnosis of infection. Routine screening and repeated HHV6 PCR may be performed in high-risk patients, such as those with low ALC. Interestingly, HHV6 reactivation in our study was not associated with an increased risk of CMV reactivation, in contrast to findings from other studies in non-HAPLO HCT settings.^5,8,40–42^ The cumulative incidence (53%) was not higher than that reported by others on HAPLO HCT without ATG or allogeneic HCT with non-HAPLO donors during the pre-letermovir era.^43–45^ These results confirm that low-dose (≤ 5 mg/kg) pre-transplant ATG can be safely added to PT-Cy in HAPLO PBSC transplantation, with respect to the risk of viral infections.

The precise impact of HHV6 reactivation on morbidity, quality of life, and mortality after HCT remains unclear. While some studies have reported an increased post-transplant mortality associated with HHV6 reactivation,^12,41,42^ others have found no detrimental effect on NRM.^8,35,39^ We did not observe any significant impact of HHV6 reactivation on RI, NRM, OS, or DFS. Additionally, the correlation between HHV6 DNA loads and clinical outcomes has yielded conflicting results. While higher HHV6 DNA loads have been associated with development of encephalitis,^46^ other authors have found no correlation with clinical signs,^47^ prediction of CMV reactivation,^16^ or patient outcomes such as OS.^8,35,39^ Interestingly, in our cohort, HHV6 DNA loads were higher in the case of clinically significant HHV6 infections (always above 10,000 IU/mL, with a median of 468,447 IU/mL). However, the level of HHV6 DNA loads was not associated with patient outcomes in terms of RI and NRM.

Our study provides deeper insights into the benefit of treating HHV6 reactivation with antiviral therapies. While ganciclovir, foscarnet, and cidofovir have demonstrated inhibitory effects on HHV6 replication *in vitro*, there are insufficient data to guide curative treatment approaches for HHV6 reactivation and clinically significant HHV6 infection, with the exception of HHV6 encephalitis.^29^ Although prophylactic or preemptive treatment approaches for HHV6 reactivation are not recommended in HCT recipients, the curative use of ganciclovir or foscarnet is probably useful in HHV6-related disease, especially in the case of encephalitis.^29,46^ In our study, a majority of patients (66%) with HHV6 reactivation received antiviral therapy, primarily due to concomitant CMV reactivation. This high proportion of patients receiving antiviral therapy might have contributed to the low incidence of clinically significant HHV6 infections. Although patients with clinically significant HHV6 infection had favorable outcomes after antiviral therapy, our study was not designed to assess the efficacy of curative antiviral approaches. It remains unknown how symptomatic patients, especially those with HHV6-related colitis or hepatitis, would have fared without any antiviral therapy. A prospective, randomized, placebo-controlled trial would be needed to address this critical question, considering the substantial toxicities associated with antiviral agents.

Other findings include a delayed platelet engraftment and an increased risk of acute GvHD in patients experiencing HHV6 reactivation. These are consistent with those from most previous studies, although the causative role of HHV6 reactivation as a trigger for acute GvHD remains a subject of debate.^5–9,12,35,39,42,48–50^ The main limitations of our study are related to its retrospective nature, despite the systematic monitoring of HHV6 with PCR. Our cohort consisted of patients with heterogenous underlying diseases and conditioning regimens, which may introduce confounding factors affecting the incidence of HHV6 reactivation and patient outcomes. Immune reconstitution is influenced not only by donor type (HAPLO or non-HAPLO), source of hematopoietic cells (PBSC, BM, or UCBT), and GvHD prophylaxis (PT-Cy, ATG), but also by pre-transplant factors such as hematologic disease, treatments administered, conditioning regimens, and post-transplant factors including GvHD, infections, status of the gut microbiota, and the therapies used to manage post-transplant complications.

In conclusion, HHV6 reactivation is common after HAPLO HCT with PT-Cy, although the incidence is lower compared to UCBT or HAPLO HCT with *ex-vivo* T-cell depleted grafts. Importantly, the incidence of clinically significant HHV6 infections remained low, even with the addition of ATG to PT-Cy. The main risk factor for HHV6 reactivation was a low ALC at day +30 after HCT. While HHV6 reactivation did not significantly impact survival outcomes, it was associated with delayed platelet recovery and an increased incidence of acute GvHD, as described in non-HAPLO HCT. Overall, our findings show that combining ATG and PT-Cy in unmanipulated HAPLO PBSC transplantation is a safe and valid approach that does not increase the risk of HHV6 reactivation or infection.

### Authorship statement

AP provided clinical care, recruited patients, performed statistical analyses, and wrote the manuscript. TM provided clinical care, collected, assembled, and analyzed data, and wrote the manuscript. JGM performed biological analyses and provided virologic expertise and support in the manuscript writing. FM, EB, AB, ZVW, TL, RB, SS, AC, NS, AB, AV, OL, and MM provided clinical care and commented on the manuscript. All authors approved the submission of the final version of the manuscript for publication purposes.

### Competing Interests

FM reports lecture honoraria from Therakos/Mallinckrodt, Sanofi, JAZZ Pharmaceuticals, Gilead, Novartis, and Bristol Myers Squibb, all outside the submitted work. MM reports grants and lecture honoraria from Janssen, Sanofi, Maat Pharma and JAZZ Pharmaceuticals, lecture honoraria from Celgene, Amgen, BMS, Takeda, and Pfizer, grants from Roche, all outside the submitted work. RD reports research funding from Ligue contre le Cancer, Arthur Sachs, Monahan Foundation, Servier Foundation, Philippe Foundation, DCP AP-HP, honoraria from Novartis and Takeda, non-financial support from Kite Pharma / Gilead, all outside the submitted work. The other authors declare no competing financial interests.

## Supplementary Material

Supplementary Material
