# Supplementary material for: Human herpesvirus type 6 reactivation after haploidentical hematopoietic cell transplantation with post-transplant cyclophosphamide and antithymocyte globulin: risk factors and clinical impact
Source: Clin Hematol Int. 2024 Feb 2;6(1):26–38. doi: 10.46989/001c.92525 (PMC11087003; doi:10.46989/001c.92525)
Supplement: Supplementary Material [file chi_2024_6_1_92525_193179.docx]

**Human herpesvirus type 6 reactivation after haploidentical hematopoietic cell transplantation with post-transplant cyclophosphamide and antithymocyte globulin: risk factors and clinical impact**

**Supplementary Material**

**Table S1. Univariate analyses**

|  | **Neutrophil engraftment at day +30**  % (95% CI) | **Platelet engraftment at day +180**  % (95% CI) | **Acute grade II-IV GvHD at day +100**  % (95% CI) | **NRM**  **at day +100**  % (95% CI) | **Relapse incidence**  **at 3 years**  % (95% CI) | **DFS**  **at 3 years**  % (95% CI) | **OS**  **at 3 years**  % (95% CI) |
| --- | --- | --- | --- | --- | --- | --- | --- |
| **HHV6 reactivation** | 91 (82-100) | 73 (62-86) | 39 (28-55) | 11 (5-23) | 22 (13-36) | 44 (30-58) | 50 (36-63) |
| **No HHV6 reactivation** | 96 (91-100) | 82 (71-95) | 9 (4-23) | 14 (6-29) | 39 (26-57) | 43 (27-59) | 52 (36-68) |
| *p value* | 0.32 | 0.26 | < 0.001 | 0.11 | 0.06 | 0.73 | 0.68 |
| **Age**  ≤ 51 years | 96 (90-100) | 78 (67-91) | 32 (21-48) | 10 (4-23) | 38 (27- 55) | 34 (22-46) | 41 (18-44) |
| **Age**  > 51 years | 92 (84-100) | 76 (65-89) | 20 (11-35) | 14 (7-28) | 20 (11-35) | 54 (40-68) | 62 (50-74) |
| *p value* | 0.59 | 0.59 | 0.12 | 0.86 | 0.04 | 0.05 | 0.07 |
| **PBSC** | 95 (89-100) | 79 (70-89) | 25 (17-37) | 15 (8-25) | 25 (17-37) | 46 (34-58) | 55 (43-67) |
| **BM** | 92 (80-100) | 71 (54-93) | 29 (15-55) | 4 (1-30) | 42 (26-68) | 38 (20-56) | 41 (21-61) |
| *p value* | 0.46 | 0.33 | 0.7 | 0.34 | 0.09 | 0.6 | 0.46 |
| **Male** | 94 (87-100) | 79 (69-90) | 26 (17-40) | 8 (4-19) | 26 (17-40) | 46 (34-58) | 53 (39-67) |
| **Female** | 95 (87-100) | 74 (61-90) | 26 (15-44) | 18 (9-36) | 33 (21-53) | 41 (25-57) | 49 (33-65) |
| *p value* | 0.57 | 0.89 | 0.93 | 0.94 | 0.4 | 0.39 | 0.52 |
| **DRI** (low-intermediate) | 96 (90-100) | 84 (75-93) | 25 (17-38) | 8 (3-18) | 21 (13-34) | 54 (42-66) | 60 (48-72) |
| **DRI** (high-very-high) | NA | 64 (49-83) | 27 (15-48) | 21 (11-42) | 46 (31-67) | 24 (8-40) | 33 (17-49) |
| *p value* | 0.47 | 0.02 | 0.61 | 0.47 | 0.006 | < 0.001 | 0.002 |
| **AML** | 94 (87-100) | 77 (65-89) | 28 (18-43) | 16 (8-30) | 22 (13-37) | 44 (30-58) | 51 (37-65) |
| **Other disease** | 94 (87-100) | 78 (66-91) | 25 (15-40) | 8 (3-21) | 37 (26-54) | 43 (29-57) | 51 (37-65) |
| *p value* | 0.40 | 0.9 | 0.76 | 0.06 | 0.12 | 0.65 | 0.6 |
| **Previous lines** (0-1) | 92 (84-100) | 81 (70-94) | 25 (15-41) | 6 (2-19) | 23 (14-39) | 54 (40-68) | 61 (47-75) |
| **Previous lines** (≥ 2) | NA | 73 (62-87) | 27 (17-42) | 17 (10-32) | 35 (24-51) | 34 (20-48) | 42 (28-56) |
| *p value* | 0.82 | 0.61 | 0.98 | 0.33 | 0.17 | 0.03 | 0.02 |
| **CR** | 98 (93-100) | 85 (75-96) | 22 (13-38) | 9 (3-22) | 26 (16-43) | 54 (40-68) | 67 (53-81) |
| **No CR** | 91 (83-99) | 70 (59-84) | 30 (20-45) | 15 (8-28) | 32 (21-47) | 35 (21-49) | 38 (24-52) |
| *p value* | 0.12 | 0.01 | 0.43 | 0.12 | 0.41 | 0.02 | 0.003 |
|  | **Neutrophil engraftment at day +30**  % (95% CI) | **Platelet engraftment at day +180**  % (95% CI) | **Acute grade II-IV GvHD at day +100**  % (95% CI) | **NRM**  **at day +100**  % (95% CI) | **Relapse incidence**  **at 3 years**  % (95% CI) | **DFS**  **at 3 years**  % (95% CI) | **OS**  **at 3 years**  % (95% CI) |
| **RIC** | 94 (84-100) | 77 (62-94) | 17 (7-38) | 10 (3-30) | 30 (17-53) | 40 (22-58) | 53 (35-71) |
| **MAC** | 96 (88-100) | 93 (82-100) | 33 (19-58) | 4 (1-26) | 19 (8-42) | 63 (45-81) | 70 (54-86) |
| **Sequential conditioning** | NA | 67 (54-84) | 28 (17-45) | 19 (10-35) | 35 (23-53) | 35 (21-49) | 37 (21-53) |
| *p value* | 0.32 | 0.02 | 0.31 | 0.47 | 0.3 | 0.04 | 0.03 |
| **Year of HCT**  ≤ 2015 | 94 (87-100) | 71 (60-85) | 31 (20-47) | 10 (4-22) | 33 (22-49) | 40 (26-54) | 46 (32-60) |
| **Year of HCT**  > 2015 | 94 (86-100) | 83 (73-95) | 21 (12-36) | 15 (7-29) | 25 (15-41) | 48 (34-62) | 58 (44-72) |
| *p value* | 0.74 | 0.29 | 0.18 | 0.91 | 0.43 | 0.64 | 0.46 |
| **Patient CMV serostatus** negative | 97 (90-100) | 82 (70-97) | 27 (15-47) | 3 (0-21) | 30 (18-51) | 55 (37-73) | 61 (41-81) |
| **Patient CMV serostatus** positive | 92 (86-99) | 74 (64-86) | 26 (17-39) | 17 (10-29) | 28 (20-42) | 38 (26-50) | 45 (33-57) |
| *p value* | 0.05 | 0.33 | 0.93 | 0.04 | 0.9 | 0.03 | 0.03 |
| **CMV reactivation** | 92 (83-100) | 79 (69-91) | 25 (15-40) | 11 (5-24) | 21 (12-35) | 49 (35-63) | 57 (43-71) |
| **No CMV reactivation** | 96 (91-100) | 75 (63-89) | 28 (17-44) | 13 (6-27) | 39 (27-56) | 38 (24-52) | 44 (28-60) |
| *p value* | 0.60 | 0.21 | 0.78 | 0.5 | 0.07 | 0.38 | 0.39 |
| **EBV increased viral load** | 88 (78-99) | 80 (70-91) | 27 (18-41) | 3 (1-13) | 22 (14-36) | 56 (42-70) | 60 (48-72) |
| **No EBV increased viral load** | 98 (94-100) | 72 (60-89) | 24 (14-42) | 24 (14-42) | 39 (26-58) | 27 (13-41) | 39 (27-51) |
| *p value* | 0.09 | 0.33 | 0.79 | 0.1 | 0.04 | < 0.001 | 0.04 |

*Abbreviations:* GvHD, graft-versus-host disease, NRM: non-relapse mortality, DFS: disease-free survival, OS: overall survival, HHV6: human herpesvirus type 6, PBSC: peripheral blood stem cells, BM: bone marrow, DRI: disease risk index, AML: acute myeloid leukemia, CR: complete remission at transplant, RIC: reduced intensity conditioning, MAC: myeloablative conditioning, HCT: hematopoietic cell transplantation, CMV: cytomegalovirus, EBV: Epstein-Barr virus, NA: not applicable.
